# A middle-aged female with recurrent sinopulmonary infections: a case report

**DOI:** 10.1186/1752-1947-2-117

**Published:** 2008-04-21

**Authors:** Umesh C Yalavarthy, Mukta Panda

**Affiliations:** 1 Department of Medicine, University of Tennessee, College of Medicine-Chattanooga, Chattanooga, TN 37403, USA

## Abstract

**Introduction:**

Common variable immunodeficiency (CVID) is a form of severe antibody deficiency with an estimated prevalence of 1 in 25,000 to 1 in 100,000. The disorder apparently results from currently undefined immune deregulations resulting in failed B-cell differentiation with impaired secretion of immunoglobulins. It has a broad range of clinical symptoms including recurrent infections of the respiratory tract, chronic lung disease, autoimmune diseases, liver and gastrointestinal disorders, granulomatous infiltrations, lymphoma and solid tumors.

**Case presentation:**

A 42-year-old Caucasian female presented with a one-day history of high-grade fever and productive cough associated with retrosternal chest pain. The patient had been discharged one week prior after a prolonged stay in an intensive care unit with multiorgan failure requiring temporary hemodialysis for two weeks secondary to sepsis. Past medical history was significant for chronic obstructive pulmonary disease, recurrent pneumonias and recurrent sinus infections since adolescence. She had a temperature of 99.8°F, was tachycardic (137/min), tachypneic (26/min) with a blood pressure of 109/59 mmHg and oxygen saturation of 88% on 2 l/min nasal oxygen. Physical examination was significant for bibasilar rhonchi. Laboratory data were significant for leukocytosis of 15,700/mm^3^. Chest X-ray demonstrated bibasilar infiltrates. The patient was started on intravenous levofloxacin and vancomycin, and sputum gram stain and cultures were performed. Given the patient's recurrent respiratory infections, an underlying immunologic disorder was considered. Work-up revealed immunoglobulin A (IgA) 11 mg/dl (normal 70–400 mg/dl), immunoglobulin M (IgM) 2 mg/dl (normal 40–230 mg/dl) and IgG 53 mg/dl (normal 700–1,600 mg/dl). The patient was diagnosed with CVID and started on intravenous immunoglobulin. She was initially started on a four-week regimen of intravenous immunoglobulin, which was later switched to a three-week regimen as the patient had respiratory infections on the four-week regimen. She remained asymptomatic on a three times/week intravenous immunoglobulin regimen.

**Conclusion:**

This case emphasizes the need for a high index of clinical suspicion for CVID in patients presenting with recurrent sinopulmonary infections. Although intravenous immunoglobulin provides improvement in these patients, early diagnosis is the key to preventing significant morbidity and mortality and improving prognosis.

## Introduction

Common variable immunodeficiency (CVID) is a form of severe antibody deficiency with an estimated prevalence of 1 in 25,000 to 1 in 100,000. The disorder results from failed B-cell differentiation with impaired secretion of immunoglobulins. It has a broad range of clinical manifestations including recurrent infections of the respiratory tract and chronic lung disease, autoimmune diseases, gastrointestinal disorders, granulomatous infiltrative diseases, lymphoma and solid tumors. We report a case of a 42-year-old Caucasian female who had presented with classical symptoms of CVID over a period of several years and review the various clinical manifestations, diagnosis and treatment options for CVID.

## Case presentation

A 42-year-old Caucasian female presented with a history of high-grade fever and yellow productive cough for one day. The cough was associated with pleuritic chest pain and shortness of breath at rest. The patient described chronic dyspnea for approximately 15 years limiting her ability to perform household activities. A review of systems was significant for chronic fatigue and weakness for 15 years, and chronic loose stools with occasional constipation for 10 years. The patient had been transferred from the hospital to a rehabilitation facility one week prior following a prolonged stay in the intensive care unit with multiorgan failure secondary to septic shock. She required ventilatory support for two weeks and temporary hemodialysis for approximately two weeks secondary to acute kidney injury. She was discharged in a stable condition.

Past medical history included asthma for 15 years, chronic obstructive pulmonary disease for 5 years, recurrent cough and sinus infections since adolescence requiring antibiotics, severe ear infection requiring emergent mastoidectomy 4 years prior, gastroesophageal reflux disease, irritable bowel syndrome for 10 years, migraine headaches and bipolar disorder for 2–3 years, and tonsillectomy as a child. She had a normal mammogram 4 years prior and a normal colonoscopy approximately 10 years prior, which was performed as part of a work-up for irritable bowel syndrome. She admitted to a 20 pack year smoking history, but denied any alcohol or illicit drug abuse. Medications included levalbuterol, montelukast, fluticasone/salmeterol for 10–15 years, lansoprazole and loratidine/pseudoephedrine as required for a few years, and hydrocodone, quetiapine fumarate and topiramate for approximately 3 years.

On examination, she appeared chronically ill and anxious. She had a temperature of 99.8°F, was tachycardic (137/min), tachypneic (26/min) with a blood pressure of 109/59 mmHg and was saturating 88% on 2 l/min nasal oxygen. Physical examination was significant for bibasilar rhonchi and a central venous catheter in the left internal jugular vein, which had been placed for hemodialysis. She had no clubbing or nasal polyps. The remainder of the physical examination was within normal limits.

Her white blood cell (WBC) count was elevated at 15,800/mm^3^; this was 8,000/mm^3 ^at discharge. Her blood urea nitrogen (BUN) and creatinine levels were slightly elevated at 24/1.6 mg/dl; however, these were on a downward trend since discharge (29/2.4 mg/dl). Chest X-ray was significant for improving bibasilar infiltrates when compared with a previous chest X-ray at the time of discharge. Cardiac enzymes were normal and arterial blood gas (ABG) showed a pH of 7.5, PC0_2 _of 32 mmHg, PO_2 _of 48 mmHg and HCO_3 _of 25 mEq/l on 2 l/min nasal oxygen. The A-a gradient was markedly increased at 104 mmHg. Computerized tomography (CT) of the chest following the pulmonary embolism protocol did not reveal any evidence of pulmonary embolism. Sputum Gram stain with cultures and blood cultures were sent for analysis and she was started on intravenous levofloxacin and vancomycin.

**Figure 1:**
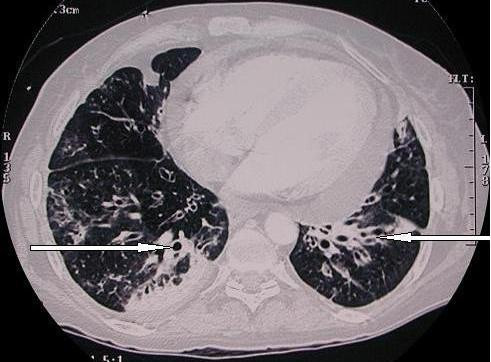
**CT scan of the chest**. Bronchial wall thickening and dilatation predominantly in the lower lung zones consistent with bronchiectasis can be seen.

A high-resolution CT thorax scan without contrast was carried out for better delineation of lung parenchyma and was significant for bronchial wall thickening and dilatation predominantly in the lower lung fields consistent with bronchiectasis (Figure 1).

A CT scan of the sinuses revealed chronic maxillary sinusitis. The patient showed clinical improvement by the next day and the blood culture did not have any growth at 72 hours. Sputum culture showed moderate growth of *Streptococcus pneumoniae*.

Given the patient's recurrent respiratory infections since adolescence and bronchiectasis, a wide differential diagnosis was considered to include genetic conditions such as cystic fibrosis, immotile cilia syndrome, alpha 1 antitrypsin deficiency and immunodeficiency. A work-up was initiated which revealed a negative sweat chloride test and normal alpha 1 antitrypsin levels. Human immunodeficiency virus (HIV) screening test was negative. Ig levels were significantly reduced: IgA 11 mg/dl (normal 70–400 mg/dl), IgM 2 mg/dl (normal 40–230 mg/dl) and IgG 53 mg/dl (normal 700–1,600 mg/dl). The presumptive diagnosis of CVID was confirmed by an inadequate antibody response to pneumococcal vaccine and tetanus toxoid. The patient was given 400 mg/kg of intravenous immunoglobulin (IVIG). She was initially started on a four-week regimen of IVIG infusion, which was later switched to a three-week regimen as the patient had respiratory infections on the four-week regimen. Since initiation of this regimen 18 months ago, the patient has required no hospital admissions or antibiotic treatments and reported a significant improvement in quality of life.

## Discussion

CVID is a rare form of severe antibody deficiency with an incidence of 1 in 25,000 to 1 in 100,000. The mean age of diagnosis is 30 years, although there can be a delay in diagnosis by many years as demonstrated in our patient [1]. The disorder results from failed B-cell differentiation. Thus, plasma cells do not develop and immunoglobulin secretion is impaired. A number of defects of T-cell function and deficits in the memory B-cell pool have been identified, but the underlying cause of this defect remains unknown [1].

CVID has variable clinical manifestations, the most common being recurrent bacterial infections caused by encapsulated bacteria [1]. Bacterial infections commonly involve the sinuses and respiratory tract leading to sinusitis, otitis media, bronchitis and pneumonia. Chronic sinusitis and bronchiectasis are frequent complications in untreated patients leading to significant morbidity and mortality [1]. Giardiasis is a frequent infection in patients leading to chronic diarrhea. They can also have diarrhea secondary to dysgonic fermenter 3, which is unusual in immunocompetent patients [2]. They are prone to severe herpes simplex, cytomegalovirus infections of the gastrointestinal tract [3,4] and meningoencephalitis from enteroviral infection. Patients who are not receiving IVIG owing to a delay in diagnosis may develop sepsis or meningitis, which can be fatal [1]. These patients are also at higher risk of developing other autoimmune diseases such as thrombocytopenic purpura, hemolytic anemia and/or neutropenia [5]. In the largest published case series of 248 patients, a 7.7% incidence of non-Hodgkin's lymphoma (NHL) was reported [1]. Mucosal associated lymphoid tissue lymphomas, an uncommon form of NHL, can occur in these patients in the stomach or bronchial tissue [6,7]. Other uncommon manifestations include granulomatous lung disease, follicular bronchiolitis, inflammatory bowel disease, sprue-like illness, nodular lymphoid hyperplasia and lymphoid interstitial pneumonia [8].

CVID should be suspected in any patient with recurrent infections, especially of the upper or lower respiratory tract. IgG, IgA or IgM levels should be less than two standard deviations below the mean for age-adjusted standardized reference. They should also have inadequate antibody response to pneumococcal vaccine and tetanus toxoid or absent isohemagglutinins to confirm the diagnosis [9].

The mainstay of treatment is IVIG. The target trough level should be 400–500 mg/dl, which is achieved by infusing a dose of 200–400 mg/kg every three or four weeks. The dosage varies from patient to patient, and IgG levels should be checked periodically to attain a target trough level. Autoimmune and granulomatous components of this disease do not respond to treatment with IVIG. There has been recent interest in the use of tumor necrosis factor (TNF) antagonists and anti-CD20 immunomodulators in treating autoimmune and granulomatous diseases based on the dramatic improvement of some clinical manifestations documented in some case reports [10-12]. However, long-term immunomodulators should be used with extreme caution as these patients are at high risk of developing malignancies.

## Conclusion

The case presented here emphasizes the need for a high index of clinical suspicion for CVID in patients presenting with recurrent sinopulmonary infections. Although IVIG provides improvement in these patients, early diagnosis is the key to preventing significant morbidity and mortality and improving prognosis.

## Abbreviations

ABG: arterial blood gas; BUN: blood urea nitrogen; CT: computerized tomography; CVID: common variable immunodeficiency; HIV: human immunodeficiency virus; Ig: immunoglobulin; IVIG: intravenous immunoglobulin; NHL: non-Hodgkin's lymphoma; TNF: tumor necrosis factor; WBC: white blood cell count.

## Competing interests

The authors declare that they have no competing interests.

## Authors' contributions

UCY was involved in conception of the case report, review of the literature and writing the manuscript. MP performed critical analysis for intellectual content and helped to draft the manuscript. Both authors read and approved the final manuscript.

## Consent

Written informed consent was obtained from the patient for publication of this case report and accompanying images. A copy of the written consent is available for review by the Editor-in-Chief of this journal.
